# Social network and inequalities in smoking amongst school-aged adolescents in six European countries

**DOI:** 10.1007/s00038-016-0830-z

**Published:** 2016-05-12

**Authors:** Vincent Lorant, Victoria Soto Rojas, Pierre-Olivier Robert, Jaana M. Kinnunen, Mirte A. G. Kuipers, Irene Moor, Gaetano Roscillo, Joana Alves, Arja Rimpelä, Bruno Federico, Matthias Richter, Julian Perelman, Anton E. Kunst

**Affiliations:** 1 Institute of Health and Society, Université Catholique de Louvain, Clos chapelle aux champs 30/B1.30.15.05, 1200 Brussels, Belgium; 2 School of Health Sciences, University of Tampere, Tampere, Finland; 3 Department of Public Health, Academic Medical Center, University of Amsterdam, Amsterdam, The Netherlands; 4 Institute of Medical Sociology (IMS), Medical Faculty, Martin Luther University Halle-Wittenberg, Halle (Saale), Germany; 5 Department of Human Sciences, Society and Health, University of Cassino and Southern Lazio, Cassino, Italy; 6 National School of Public Health, University of Lisbon, Lisbon, Portugal; 7 Department of Adolescent Psychiatry, Pitkäniemi Hospital, Nokia, Tampere University Hospital, Tampere, Finland

**Keywords:** Smoking, Socio-economic inequalities, Adolescent, Social network

## Abstract

**Objectives:**

Smoking contributes to socio-economic health inequalities; but it is unclear how smoking inequalities emerge at a young age. So far, little attention has been paid to the role of friendship ties. We hypothesised that the combination of peer exposure and friendship social homophily may contribute to socio-economic inequalities in smoking at school.

**Methods:**

In 2013, a social network survey was carried out in 50 schools in six medium-size European cities (Namur, Tampere, Hanover, Latina, Amersfoort, and Coimbra). Adolescents in grades corresponding to the 14-to-16 age group were recruited (*n* = 11.015, participation rate = 79.4 %). We modelled adolescents’ smoking behaviour as a function of socio-economic background, and analysed the mediating role of social homophily and peer exposure.

**Results:**

Lower socio-economic groups were more likely to smoke and were more frequently exposed to smoking by their close and distant friends, compared with adolescents of higher SES. The smoking risk of the lowest socio-economic group decreased after controlling for friends smoking and social homophily.

**Conclusions:**

Smoking socio-economic inequalities amongst adolescents are driven by friendship networks.

**Electronic supplementary material:**

The online version of this article (doi:10.1007/s00038-016-0830-z) contains supplementary material, which is available to authorized users.

## Introduction

Smoking is a leading behavioural contributor to socio-economic inequalities in health. (Jha et al. 2006); already in adolescence, smoking is more frequent in lower than in higher socio-economic groups (Hanson and Chen 2007; Richter et al. 2009). Recent analyses of trends in smoking inequalities amongst adults suggest that the differences have not decreased and may even have widened in some countries (Pampel 2009; Peretti-Watel et al. 2009). A comparative cohort analysis in the US, France, and Germany showed that educational disparities in smoking have increased in younger cohorts, particularly amongst women (Pampel et al. 2014). As many smokers begin smoking in adolescence, understanding smoking inequalities amongst them would make a major contribution to explaining smoking-related health differences in adulthood (Maralani 2013).

Schools play an important role in early social stratification, as adolescents’ future socio-economic opportunities depend on their curricular achievement and tracks. School is also a major source of tie formation, accounting for around 75 % of their friendship ties (Witkow and Fuligni 2010). These social ties may be a major driver of smoking through a mechanism known as peer effect: smokers are more likely to befriend smokers (Ennett et al. 2006; Mercken et al. 2009). Adolescents’ friendship ties, moreover, are socially homophilous: they prefer to mix with adolescents of a similar social background. This social homophily may magnify initial inequalities associated, e.g. with parental smoking, which varies according to social background. The literature has not fully explained the emergence of smoking inequalities in adolescence and little research has been carried out into the contribution of peers to unequal smoking distribution across socio-economic groups. Here, we hypothesised that the combination of peer effect and social homophily may contribute to smoking inequalities at school, a theory known as network-induced inequality (Dimaggio and Garip 2011; Lorant and Bhopal 2011). We report here the results of the SILNE survey, which assesses how smoking inequalities result from social ties at school.

### Theory of network-induced inequalities in smoking

According to the theory of network-induced inequalities, socio-economic inequalities in adolescent smoking arise when two conditions are met: smoking is an interdependent behaviour and social ties are socially homophilous (Dimaggio and Garip 2011).

### Smoking by peers

Amongst adolescents, smoking is an interdependent behaviour (Mercken et al. 2007). Non-smoking adolescents are more likely to become smokers if they are part of a smoking group of friends than part of a non-smoking group and to quit smoking if they are part of a non-smoking group (Seo and Huang 2012). The behavioural rationale for this interdependence includes externalities: the benefits or social cost of smoking depends on others taking up the behaviour. Indeed, smoking helps to define the group frontiers, creates social cohesion and leads to commitment amongst members (Stewart-Knox et al. 2005). The negative externalities include passive smoking and social disapproval because of smoking (Nyborg and Rege 2003).

### Social homophily

Social ties are not formed randomly: they are more likely to be created or maintained between individuals who share similar attributes such as gender, socio-economic status, or ethnicity/race, a preference called homophily (Rivera et al. 2010; Steglich et al. 2012). Homophilous social relationships amongst adolescents may magnify smoking inequalities between socio-economic groups: if one SES group has a higher parental smoking prevalence, then social homophily may concentrate the higher smoking prevalence in that group whilst keeping the other groups insulated from it (Avenevoli and Merikangas 2003).

**Fig. 1:**
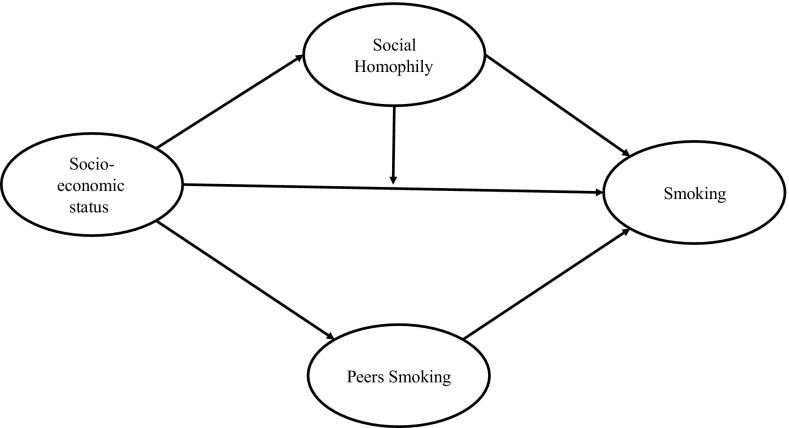
Inequalities in smoking: conceptual model

This paper investigates the role of social ties in socio-economic smoking differences in the school context. Our hypothesis is that socio-economic status affects adolescents’ smoking partly as a result of the combination of peer effect and social homophily (Fig. 1). We addressed the following two questions:

- What is the risk of smoking and exposure to friends’ smoking according to socio-economic status amongst school-aged adolescents?
- To what extent socio-economic differences in the risk of smoking are explained by having similar peers in terms of smoking and socio-economic status?

## Methods

### Setting

The survey design and instruments have been presented elsewhere (Lorant et al. 2015). SILNE is a school-based social network survey of adolescents in the grades corresponding to 14- to 16-year-olds, in six European countries: three countries with greater socio-economic differences between low and high educational achievers (Belgium, Germany, and the Netherlands) and three with little or no difference (Finland, Italy, and Portugal) (OECD 2007). In each country, a city of medium size with a median income (nationally or regionally) and a mainly tertiary economic sector was selected: Namur (Belgium), Tampere (Finland), Latina (Italy), Amersfoort (Netherlands), Hanover (Germany), and Coimbra (Portugal).

### Design

SILNE applied a whole-network approach (Knoke et al. 2008), with the boundary of the network defined as the two grades corresponding to 14- to 16-year-olds, the group most relevant for the transition to smoking (Dierker et al. 2012). In these grades, all registered teenagers were invited.

The survey took place in 2013. It was a self-completed paper and pencil questionnaire (http://silne.ensp.org/instruments_wp5/), distributed during school hours by two researchers (in Finland by teachers). After the research objectives were explained, the students were requested to participate and were given the school directory and the questionnaire. It took on average 30 min to complete. In Finland, access to the directories was not granted: the written names were written and checked afterwards with school secretaries.

### Sample

In each city, we aimed to collect 1800 questionnaires from 6 to 8 schools stratified into two groups (lower and higher SES schools). The stratification was carried out according to the information available: the type of school (Italy, Germany, the Netherlands), the socio-economic ranking of the school by the educational authorities (Belgium, Portugal), or the area’s socio-economic characteristics (Finland).

Fifty schools, out of the 163 invited, participated. Schools refused to participate for different reasons; but the most frequent one was the inappropriate timing of the survey given their scheduled activities, including curricular ones. The non-participating schools were replaced by schools of similar socio-economic ranking. The number of schools varied between countries from 6 in Portugal to 13 in Germany, a difference due to school size. The sample contained 13,870 students, of whom 11,015 participated, yielding a participation rate at the adolescent level of 79.4 %. Non-participants were classified into three categories: absent on the survey days (*n* = 1864), unwilling to participate (*n* = 461), and others (*n* = 65). Information was missing on one or more key items in 3.7 % of the questionnaires, so we were left with 10,604 complete records.

### Ethical review

National teams obtained ethical approval from local/national authorities (see (Lorant et al. 2015) School principals, parents, and students received leaflets, information letters, and parental consent letters according to the regulations of each country. Active parental consent was required in Italy and Germany.

### Measures

Three smoking variables were used: ever tried smoking, a regular smoker (smoking at least one cigarette/day), and a nicotine dependence score (the Stanford dependence index) (O’Loughlin et al. 2002).

Socio-economic status was measured by father’s and mother’s education, family affluence, subjective social status, parental working status, and housing ownership. Parental education was classified as low, middle, high, or unknown according to the education system of each country. The family affluence score (FAS, the number of cars in the household, the number of holidays/year, the number of computers, having an own bedroom) was computed and divided by the national average to allow for cross-country comparison (Richter et al. 2009). The McArthur scale of subjective social status (youth version) was classified into five groups (Goodman 1999). Parents’ working status (working or not the previous week) and household ownership (owner/tenant) were asked about. We also created a composite index of socio-economic status based on the number of times an adolescent was in the lowest category (hereafter, SES). For parental educational status, a missing reply was categorised as “unknown” (father: 17.5 %, mother: 14.8 %) to keep the information available on the other, completed indicators. We assigned the lowest category of the Mc Arthur when the information was missing (*n* = 371), the average family affluence scale when the FAS was missing, and when employment was missing we considered parents were working (father: *n* = 183, mother: *n* = 187).

Adolescent friendship ties were asked about with a question: “Who are your best and closest friends?” Adolescents were asked to nominate up to five friends (or alters). They were handed a student directory (with the exception of Finland, see above), which contained the names of all students enrolled in the two grades. One code was assigned to each name and respondents were asked to use the codes.

The nominations were used to build the square adjacency matrix *X* in which each element *x*_*ij*_ takes the value of 1 if *i* nominated *j* and 0 otherwise. Exposure to peer smoking was computed using the method of Valente (2010): for each adolescent we computed the number of smokers in the first (friends), second (friends of friends), and third out-degree separation sets of friends as a percentage of the number of friends in each out-degree separation set. The second- (and third-) degree separation set was computed by taking the power 2 (or 3) of the adjacency matrix (Wasserman and Faust 1994). We also computed the relative distance to smokers (the average distance to smokers, divided by the average distance to all alters), using the formula below, where *W*_*ij*_ is the social distance (number of degrees) between the individual *i* and alter *j*, *Y*_*j*_ is the smoking status of the alter *j* (0 if non-smoker, 1 for regular smoker), *s* the number of smokers, and *g* the total number of alters in the network. When two individuals were disconnected, the maximum distance in that network was used. The distinction between first-, second-, and third-degree separation is also informative for the interpretation of the results: ego may select his friends (first-degree), but may not select his friends’ friends. In addition, the second- and third-degree separation captures a bunch of direct and indirect influences, from close or more distant alters.

$$\text{Relative distance to smokers} _{i} = \frac{{{\raise0.7ex\hbox{${\mathop \sum \nolimits_{{j\text{ = 1}}}^{g} \left( {W_{ij } Y_{j} } \right)}$} \!\mathord{\left/ {\vphantom {{\mathop \sum \nolimits_{{j\text{ = 1}}}^{g} \left( {W_{ij } Y_{j} } \right)} s}}\right.\kern-0pt} \!\lower0.7ex\hbox{$s$}}}}{{{\raise0.7ex\hbox{${\mathop \sum \nolimits_{{j\text{ = 1}}}^{g} W_{ij} }$} \!\mathord{\left/ {\vphantom {{\mathop \sum \nolimits_{{j\text{ = 1}}}^{g} W_{ij} } g}}\right.\kern-0pt} \!\lower0.7ex\hbox{$g$}}}}$$

In addition, we computed the number of household members who were smokers.

The Coleman index of homophily was computed. This measures the propensity of an individual or group to create ties to the members of the same group (here, the same parental education group) (Bojanowski and Corten 2014). The index ranges from −1 (perfect heterophily: all ties external) to 1 (perfect homophily: all ties internal), 0 when the observed number of within-group ties is equal to the expected number of within-group ties under random network. The Coleman index was computed at the individual level and we presented the index for parental education to avoid collinearity with SES.

### Data analysis

We first described adolescent smoking status according to socio-economic status variables. We ran analyses of variance of the exposure to smoking according to SES, controlling for age and sex. We then used logistic regression to model the effect of network exposure on the risk of smoking associated with socio-economic status, using four nested models. In Model 1, we regressed the SES variable on regular smoking, controlling for age group and sex; in Model 2, we added exposures to friends’ smoking; in Model 3, we added social homophily on parental education and we tested for the interaction. In Model 4, we added family smoking (adolescents are more likely to smoke and to have smoking friends if their parents smoke) (Avenevoli and Merikangas 2003). The analyses were replicated with two other outcomes: having tried smoking and the nicotine dependence index. As the dependence score is highly skewed to the right and because of over-dispersion, the index was analysed with a negative binomial regression. All analyses were estimated with country random effects to control for clustering at the country level and a network component was added in model 4. Statistical analyses were carried out with SAS 9.3.

## Results

Half of the adolescents had tried tobacco, and 16.9 % were regular smokers with an average dependence score of 2.2 (0–25) (Table 1). The adolescents were exposed to 1.4 smokers in their household. At school, adolescents were slightly socially closer to smokers (10.6°) than to all alters (11.2, *t* test = 54.9, *p* < 0.001). A total of 17.0 % of adolescents’ first-degree friends were regular smokers. On average, social ties were homophilous in terms of parental education, with an average Coleman index of 0.3, statistically different from 0 (*t* test = 41.9, *p* < 0.001) but with significant variation (STD = 0.67).

**Table 1:** Socio-demographic variables, smoking status and network exposures, international survey of adolescents, 2013: percentages and numbers

			% or mean (std)	Number
City, Country (%)				
Namur, Belgium			19.0	2018
Tampere, Finland			13.6	1443
Hanover, Germany			12.9	1373
Latina, Italy			19.2	2031
Amersfoort, The Netherlands			17.6	1862
Coimbra, Portugal			17.7	1877
Gender (%)				
Female			52.2	5531
Male			47.8	5073
Age (years)			15.2 (1.0)	
Father’s education (%)				
Low			21.5	2279
Medium			31.9	3379
High			29.1	3088
Other unknown			17.5	1858
Mother’s education (%)				
Low			17.9	1901
Medium			35.7	3788
High			31.5	3341
Other-unknown			14.8	1574
Subjective socio-economic ranking (%)				
5 or less			22.8	2423
6			18.7	1980
7			27.5	2918
8			21.1	2241
9–10			9.8	1042
Father not working last week (%)			90.5	9601
No				
Yes			9.5	1003
Mother not working last week (%)				
No			80.6	8552
Yes			19.4	2052
Family affluence ratio (%)				
≤60 %			7.5	795
61–90 %			28.9	3063
91–120 %			34.5	3656
>120 %			29.1	3090
House/flat ownership (%)				
Owner			81.7	8661
Tenant-other			18.3	1943
Number of lowest socio-economic categories (%)				
0			32.6	3454
1			28.9	3060
2			19.3	2048
3			11.6	1232
4			5.5	586
5 or more			2.1	224
Smoking status				
Tried smoking (%)			46.6 (47.9)	10,604
Regular smoker (%)			16.9 (36.02)	10,604
Standford nicotine dependence (score, 0–25)			2.2 (4.6)	10,604
Exposure to smoking				
Distance to all alters (°)			11.2 (4.3)	10,199
Distance to smoking alters (°)			10.6 (4.5)	10,196
Relative distance to smokers (%)			94.0 (12.2)	10,196
Regular smokers in degree 1 (%)			17.0 (26.5)	10,196
Regular smokers in degree 2 (%)			17.8 (22.9)	10,196
Regular smokers in degree 3 (%)			17.5 (20.8)	10,196
Smokers in household (numbers)			1.4 (1.3)	10,604
Coleman index of Homophily (−1, 1)				
Across parental education			0.3 (0.67)	10,196

### Socio-economic status and smoking

On all indicators, the lowest socio-economic group had the highest prevalence of having tried smoking and regular smoking and had a higher dependence score (Table 2). Those whose fathers had a low level of education smoked more often than those whose fathers had a high level of education. Adolescents whose fathers had not worked the previous week were more likely to be smokers than those whose fathers had worked. We found a dose–response relationship for mostly all SES variables: the higher the socio-economic status, the lower the smoking prevalence.

**Table 2:** Adolescent smoking behaviour by socio-economic group, International survey of adolescents, 2013: percentage

Socio-economic status	Tried smoking			Regular smoking			Dependence (score)	*F* test	
	(%)	*χ* ^2^		(%)	*χ* ^2^				
Lowest socio-economic categories (number)									
0 (high)	42.0	68.3	<0.001	14.5	53.0	<0.001	1.9	22.0	<0.001
1	46.0			16.0			2.3		
2	48.4			18.4			2.6		
3	51.0			20.6			3.1		
4	55.3			23.5			3.3		
5 (low)	59.5			24.8			4.0		
Family affluence (% of the national mean)									
≤60 % (low)	52.4	19.4	<0.001	23.1	13.0	<0.001	3.6	20.2	<0.001
61–90 %	49.0			18.3			2.5		
91–120 %	44.0			14.7			2.2		
>120 % (high)	45.1			17.1			2.3		
Father’s educational status									
Low	54.3	85.5	<0.001	21.8	38.4	<0.001	3.0	31.2	<0.001
Medium	50.0			18.6			2.6		
High	40.4			13.0			1.8		
Other-unknown	43.2			16.5			2.5		
Mother’s educational status									
Low	53.6	74.9	<0.001	20.8	34.1	<0.001	2.9	28.6	<0.001
Medium	50.0			19.0			2.7		
High	41.1			13.9			1.8		
Other-unknown	43.0			15.7			2.4		
Subjective socio-economic ranking (decile)									
5 or less (low)	50.7	47.2	<0.001	20.2	24.3	<0.001	3.0	16.6	<0.001
6	48.6			17.3			2.4		
7	46.3			16.4			2.3		
8	43.0			15.1			1.9		
9–10 (high)	40.3			14.8			2.2		
Father’s working status									
Working last week	45.9	8.0	0.005	16.6	8.5	0.003	2.4	17.0	<0.001
Not working last week	50.8			20.4			3.0		
Mother’s working status									
Working last week	46.5	0.2	0.649	16.9	0.2	0.674	2.4	2.1	0.151
Not working last week	45.9			17.3			2.6		
Housing tenure									
Owner	45.8	8.7	0.003	16.3	9.1	0.003	2.3	41.6	<0.001
Tenant-other	49.5			19.2			3.0		

### Exposure to smoking amongst friends

Overall, lower SES was significantly associated with a higher exposure to regular smoking: e.g. in the lowest SES group, 23.1 % of first-degree friends smoked, compared to 16.5 % in the highest SES group (Table 3). This linear difference was observed for one, two, and three degrees of separation in the friendship network. The association between SES and exposure to smoking was slightly weakened when moving from the set of first-degree friends (difference of 6.6 %) to the set of second-degree friends (5.2 %) and the set of third-degree friends (4.9 %). The results were broadly robust across the different socio-economic variables (see supplementary tables). Exposure to household smoking also displayed a similar and consistent pattern: adolescents with the lowest SES were, on average, living in households with 1.8 smokers compared with 1.2 for adolescents with the highest SES.

**Table 3:** Exposure to regular smoking and social homophily in the adolescent school and parental network, by socio-economic groups, international survey amongst adolescents, 2013

SE groups	Exposure to regular smoking in 1st-degree friends (%)			Exposure to regular smoker in 2nd-degree friends (%)			Exposure to regular smoker in 3rd-degree friends (%)			Relative distance to regular smoker (%)			Smoking members in household (number)			Coleman index of homophily^a^ (−1, 1)		
	%	*F* test	*P*	%	*F* test	*P*	%	*F* test	*P*	%	*F* test	*P*	Number	*F* test	*P*	Index	*F* test	*P*
Lowest socio-economic categories (number)		15.9	<0.001		18.8	<0.001		15.7	<0.001		1.3	0.273		26.2	<0.001		108.9	<0.001
0	16.5			17.1			16.6			93.6			1.2			0.47		
1	16.7			18.2			18.2			94.2			1.3			0.30		
2	18.4			17.8			17.6			94.3			1.5			0.19		
3	17.9			19.0			19.2			94.0			1.6			0.09		
4	19.5			22.0			19.7			93.8			1.6			0.06		
5	23.1			22.3			21.5			93.4			1.8			0.01		

Results of the analysis of variance controlled for age and sex

^a^On parental education

There was a strong association between SES and homophily: friendship ties amongst adolescents with the highest SES were strongly homophilous (Coleman index = 0.47), whereas adolescents with the lowest SES were neither homophilous nor heterophilous (Coleman index = 0.01).

Logistic regression (Table 4) displayed an increasing risk of regular smoking as socio-economic status decreased (Model 1). The odds ratio (OR) increased by 26 % for each 10 % increase of smoking prevalence amongst first-degree friends and by 22 % for each 10 % increase amongst second-degree friends. The higher the relative distance to smokers, the lower the OR of smoking (Model 2). Being homophilous regarding parental education led to a lower OR (OR = 0.88, Model 3). The result of a test of interaction between homophily and SES was not significant (Wald *χ* ^2^ = 0.50, *p* = 0.47). The OR of smoking associated with low-SES categories decreased in Model 2 compared with Model 1, as well as in Model 3 compared with Model 2, particularly for adolescents in the two lowest SES categories. In Model 4, we controlled for the number of smoking household members and a network random coefficient: this had some influence on the odds ratio of exposure to smoking amongst degree 1 friends (OR = 1.26, Model 3; OR = 1.21 Model 4) or on relative distance to smokers (OR = 0.79, Model 3 and OR = 0.54, Model 4). Interestingly, parental educational homophily became less important and with borderline statistical significance in Model 4, suggesting that homophily affects adolescents’ smoking status by passing on parental behaviour.

**Table 4:** Effect of exposure to smoking on regular smoking: odds ratio from the logistic regressions, international survey of adolescents, 2013

	Model 1		Model 2		Model 3		Model 4	
	OR	95 % CI	OR	95 % CI	OR	95 % CI	OR	95 % CI
Number of lowest socio-economic categories (ref = none)								
1	1.09	(0.95–1.25)	1.10	(0.94–1.29)	1.08	(0.91–1.26)	1.02	(0.86–1.20)
2	1.21	(1.04–1.41)*	1.19	(1.00–1.43)*	1.15	(0.97–1.38)	1.00	(0.82–1.21)
3	1.29	(1.08–1.54)**	1.24	(1.01–1.53)*	1.19	(0.96–1.46)	0.96	(0.76–1.20)
4	1.44	(1.14–1.82)**	1.28	(0.97–1.70)	1.23	(0.93–1.63)	0.93	(0.71–1.20)
5	1.52	(1.08–2.14)*	1.15	(0.76–1.75)	1.10	(0.73–1.67)	1.02	(0.86–1.20)
Exposure to smoking and homophily								
Exposure to regular smoking degree 1 (10 %^§^)			1.26	(1.24–1.29)***	1.26	(1.24–1.29)***	1.21	(1.19–1.24)***
Exposure to regular smoking degree 2 (10 %^§^)			1.22	(1.18–1.25)***	1.22	(1.18–1.25)***	1.18	(1.15–1.22)***
Exposure to regular smoking degree 3 (10 %^§^)			0.97	(0.94–1.00)*	0.97	(0.94–1.00)*	0.93	(0.90–0.96)***
Relative distance to smoking (%)			0.79	(0.75–0.83)***	0.79	(0.75–0.83)***	0.54	(0.50–0.59)***
Parental education homophily (−1, +1)					0.88	(0.80–0.96)**	0.90	(0.82–0.99)*
Smokers in the household (number)							1.50	(1.43–1.57)***
Sociodemographics								
Age 14–15 (reference <14)	2.30	(1.26–4.19)**	1.95	(0.96–3.94)	1.90	(0.94–3.84)	1.71	(0.85–3.43)
Age 16+	6.26	(3.44–11.4)***	3.99	(1.97–8.09)***	3.87	(1.91–7.84)***	3.19	(1.58–6.43)**
Sex (reference = female)	1.13	(1.01–1.26)*	1.06	(0.94–1.20)	1.06	(0.94–1.20)	1.13	(0.99–1.29)
Country covariance component mean (std)	0.11 (0.07)		0.06 (0.04)		0.05 (0.04)		0.20 (0.17)	
Network covariance component mean (std)							0.42 (0.14)	

*** *p* < 0.001; ** *p* < 0.01; * 0.01 ≤ *p* < 0.05; the models are controlled for the variables displayed in the table

^§^10 % prevalence of regular smoking

The analysis was replicated with two other smoking outcomes. The risk of having tried smoking of the lowest SES group compared to the highest decreased from Model 1 (OR = 1.70) to Model 3 (OR = 1.41). Similar results were observed, although with smaller amplitude, with the dependence score: from OR = 2.1 (Model) 1 to OR = 1.89 (Model 3). SES remained significantly associated with the score of dependence in Model 4.

**Fig. 2:**
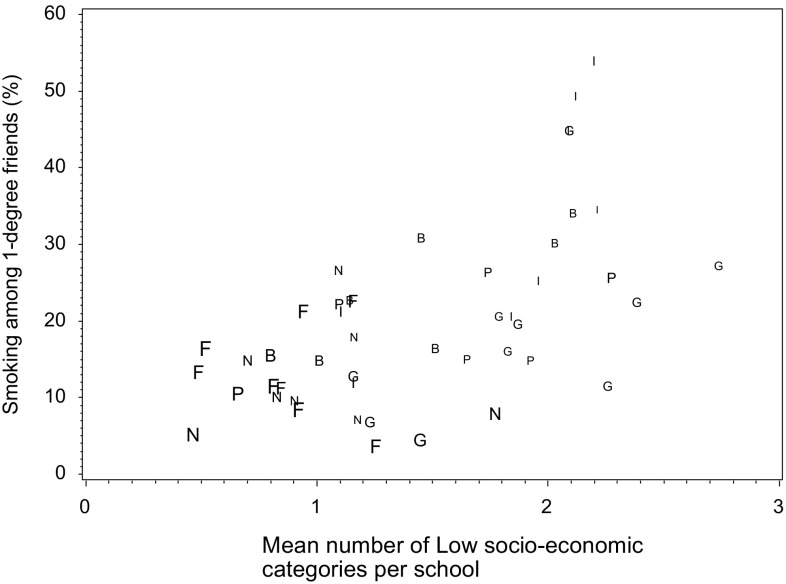
Average socio-economic status, smoking exposure and social homophily among friends, by school, International Survey of adolescents, 2013. *B* Belgium, *F* Finland, *G* Germany, *I* Italy, *N* The Netherlands, *P* Portugal. *Symbols* are proportional to the 1+ homophily score

Figure 2 displays each school according to the smoking prevalence amongst first-degree friends (*Y*-axis) and according to the mean number of lowest SES categories (*X*-axis). The figure displays an increasing prevalence of friends’ smoking according to the average number of the lowest SES categories (correlation coefficient 0.59, *p* < 0.001). Schools with higher levels of homophily (bold letters) are in the lower left quadrant: at the school level, friendship homophily on parental education is negatively associated with lower levels of friends’ smoking (correlation of −0.48, *p* < 0.001) and with higher SES (correlation of −0.69, *p* < 0.001). There is obvious heterogeneity between countries, with Finland showing higher SES values than Italy. Heterogeneity within countries was noticeable, particularly in Belgium and Germany.

## Discussion

The SILNE survey investigated whether social network exposure to smoking at school contributes to socio-economic differences in smoking. SILNE is amongst the first studies to test the theory of network-induced health socio-economic difference in smoking using cross-country social network data.

### Findings

Lower socio-economic status adolescents were more likely to have tried smoking, and to be regular smokers and dependent on nicotine than adolescents of higher socio-economic status. They were also more frequently exposed to smoking amongst their close and distant friends and amongst their household members. Further, they had a quarter of their friends smoking compared with one-sixth from the highest socio-economic group. Smoking differences across socio-economic groups were partly explained by exposure to peer smoking and to a lesser extent by social relationships homophily.

### Consistency with previous literature

Few comparable studies have investigated the role of peer smoking in socio-economic differences in smoking. In a longitudinal study, young adults of low socio-economic status were more frequently heavy smokers than young adults of higher socio-economic status, and this difference was partly associated with having more of their friends smoking (Yang et al. 2008). Amongst youth studies, a study in the Netherlands found that the higher smoking rate of 13-year-olds in the vocational track compared with adolescents in non-vocational education was associated with a higher proportion of smoking friends (Huisman and Bruggeman 2012).

Two pathways could explain why adolescent smoking behaviour is substantially related to friends’ smoking behaviour: either adolescents make friends with other smoking peers because they have a personal vulnerability to smoking (e.g. when their parents smoke) or they take up the behaviour of their existing peers. The first pathway is known as selection, whilst the second is labelled peer influence. Our cross-sectional design makes it difficult to disentangle the two and that is beyond the scope of this paper. The literature and our data provide some insights, however. Previous longitudinal research suggests that both selection and peer effect apply to smoking diffusion in a network (Mercken et al. 2009; Steglich et al. 2012). Qualitative research has also hinted that selection and influence go hand in hand (Stewart-Knox et al. 2005). Peer effect and selection may reflect different aspects of social ties and complement each other: young people tend to become similar to each other when they are in stable and reciprocal relationships, whereas new relationships are established with those with the same smoking behaviour (Fisher and Bauman 1988). A recent experimental study of the adoption of health behaviour concluded that individuals are more susceptible to influence from those who are similar to them (Centola 2011). Our study showed that adolescent smoking behaviour is related to friends’ smoking, up to the second degree of separation, and to their kin’s smoking behaviour, too. This leads to the double jeopardy of social and inter-generational transmission of smoking inequalities at school.

Adolescents with homophilous friendships (ties to adolescents of similar parental education) were less often regular smokers. This homophily also contributed slightly to socio-economic differences in smoking as the association between SES and smoking weakened when controlled for parental education homophily. This raises the question: how does homophily contribute to differences of smoking prevalence between socio-economic groups? Adolescents of higher SES groups were socially more homophilous than adolescents of lower groups, a difference which may enhance the protective effect of their SES on smoking. Heterophilous friendships bridge different sub-groups and are thus more vulnerable to different patterning of behaviour. Higher homophily in the high-SES adolescents may help to insulate then from the higher smoking in lower SES adolescents. Conversely, the low homophily in the lower SES adolescents exposes them both to the lower exposure of high-SES adolescents and to the higher smoking exposure of their own SES group: in a word, homophily rendered the smoking risk attached to SES sticky, possibly because of parental smoking status. This was somewhat supported by our finding that homophily became statistically non-significant when controlling for parental smoking status.

The moderate contribution of homophily to the association between SES and smoking may be explained by two elements. First, as shown in Table 3, adolescents from lower socio-economic status were less homophilous compared with adolescents of higher SES, possibly because of the advantage to have a broader spectrum of friendship ties when one is on the bottom of social ladder. Second, the lower value of the homophily index for adolescents of lower SES does not mean that they have as many friends from lower SES as friends from higher SES. According to the way the coleman index is computed, it means that their pattern of friendship connections matches the average distribution inside their school: in vocational schools, for example, the homophily index may be low because of the high overall proportion of low-SES adolescents in these schools. In that case, to the extent that smoking is frequent in these schools, homophily is already accounted for by the variables related to exposure to peers smoking.

### Limitations

Threats to internal validity may come from possible socio-economic differences in response patterns. We compared the numbers of questionnaires with missing information and unexpected replies (e.g. a conditional jump not complied with) in different groups. Questionnaires with a high proportion of missing answers varied unsystematically: 2 % from the low-SES groups (3+ lowest categories), 3 % in the middle (1-2 lowest categories), and 1 % in the highest (0 lowest category) (*F* = 19.6, *p* < 0.01). We also compared the correlation of self-reported friends’ smoking with first-degree percentage of peer smoking: the Spearman rank correlation was similar across the SES groups (those with no lowest SES category: 0.49; 1–2 lowest SES categories: 0.48; 3+ lowest: 0.44).

The external validity depends on whether the selected schools are representative of schools in the selected cities and whether the cities are close to the country average. Indeed, the percentage of participating schools was modest (30 %), and as smoking prevalence varies across schools, we cannot rule out a bias associated with participation at the school level. Yet, we are confident that our analysis is not very much vulnerable to this modest participation rate. First, we were not interested into smoking prevalence but into the network effects (peers’ smoking and homophily) on smoking socio-economic differences, and we also counted with a great diversity of schools, as evidenced from Fig. 2. Second, comparing our results with the HBSC2009/10 results we found that gender distribution and family affluence scores had similar distributions (Lorant et al. 2015). However, the percentages of those who had ever smoked and of daily smokers were slightly higher in SILNE than in HBSC, perhaps due to the older age group.

### Conclusions

Socio-economic inequalities in smoking are partly explained by network exposure to smoking. It may be time to consider complementary approaches, such as interventions rooted in peer influence/selection effects. Experimental studies have suggested that involving influential and homophilous peers contributes to the adoption of positive health behaviours (Thomas et al. 2013). The use of social network analysis both as analytical and intervention approach has been applied in different substance use programmes (Valente et al. 2004). One possible avenue may be to help popular adolescents either not to initiate smoking, or to quit smoking, or to persist in attempts to quit, particularly in vocational schools or in schools with a higher deprivation background.

## Electronic supplementary material

Below is the link to the electronic supplementary material.

Supplementary material 1 (DOCX 27 kb)
