# Supplementary material for: Social network and inequalities in smoking amongst school-aged adolescents in six European countries
Source: Int J Public Health. 2016 May 12;62(1):53–62. doi: 10.1007/s00038-016-0830-z (PMC5288430; doi:10.1007/s00038-016-0830-z)
Supplement: Supplementary file 1 — Supplementary material 1 (DOCX 27 kb) [file 38_2016_830_MOESM1_ESM.docx]

Social network and inequalities in smoking among school-aged adolescents in six European countries, International Journal of Public Health,

Prof. Vincent Lorant, PhD (1^*^), Dr. Victoria Soto Rojas, PhD (1), Mr. Pierre-Olivier Robert , MSSc(1), Dr. Jaana M. Kinnunen, MHS (2), Dr. Mirte A.G. Kuipers, MSc (3), Dr. Irene Moor, MSc (4), Mr. Gaetano Roscillo, MSc (5), Mrs. Joana Alves, MA (6), Prof. Arja Rimpelä, PhD (2,7), Prof. Bruno Federico, PhD (5), Prof. Matthias Richter, PhD (4), Prof. Julian Perelman, PhD (6), Prof. Anton E. Kunst, PhD (3)

(1)Institute of Health and Society, Université Catholique de Louvain, Brussels, Belgium

*Corresponding author:

[Vincent.lorant@uclouvain.be](mailto:Vincent.lorant@uclouvain.be)

Supplementary table n°1. Exposure to regular smoking and social homophily in the adolescent school and parental network, by socio-economic covariates, International survey among adolescents, 2013 *.

| SE groups | Exposure to regular smoking in 1st degree friends(%) | | | Exposure to regular smoker in 2nd degree friends(%) | | | Exposure to regular smoker in 3rd degree friends(%) | | | Relative distance to regular smoker (%) | | | Smoking members in household (nber) | | | Coleman index of homophily (-1,1) | | |
| --- | --- | --- | --- | --- | --- | --- | --- | --- | --- | --- | --- | --- | --- | --- | --- | --- | --- | --- |
|  | % | F-test | P | % | F-test | P | % | F-test | P | % | F-test | P | Nber | F-test | P | Index | F-test | P |
| **Lowest socio-economic categories (nber):** |  | 15.9 | <.001 |  | 18.8 | <.001 |  | 15.7 | <.001 |  | 1.3 | 0.273 |  | 26.2 | <.001 |  | 363.6 | <.001 |
| 0 | 16.5 |  |  | 17.1 |  |  | 16.6 |  |  | 93.6 |  |  | 1.2 |  |  | 0.60 |  |  |
| 1 | 16.7 |  |  | 18.2 |  |  | 18.2 |  |  | 94.2 |  |  | 1.3 |  |  | 0.51 |  |  |
| 2 | 18.4 |  |  | 17.8 |  |  | 17.6 |  |  | 94.3 |  |  | 1.5 |  |  | 0.25 |  |  |
| 3 | 17.9 |  |  | 19.0 |  |  | 19.2 |  |  | 94.0 |  |  | 1.6 |  |  | -0.05 |  |  |
| 4 | 19.5 |  |  | 22.0 |  |  | 19.7 |  |  | 93.8 |  |  | 1.6 |  |  | -0.24 |  |  |
| 5 | 23.1 |  |  | 22.3 |  |  | 21.5 |  |  | 93.4 |  |  | 1.8 |  |  | -0.21 |  |  |
| **Family affluence (ratio):** |  | 10.7 | <.001 |  | 16.0 | <.001 |  | 22.2 | <.001 |  | 8.7 | <.001 |  | 23.6 | <.001 |  | 254.7 | <.001 |
| <=60% | 19.3 |  |  | 20.1 |  |  | 19.8 |  |  | 93.1 |  |  | 1.6 |  |  | -0.02 |  |  |
| 61-90% | 17.4 |  |  | 18.5 |  |  | 19.0 |  |  | 95.0 |  |  | 1.5 |  |  | -0.09 |  |  |
| 91%-120% | 16.6 |  |  | 17.0 |  |  | 16.9 |  |  | 93.8 |  |  | 1.3 |  |  | 0.25 |  |  |
| >120% | 17.8 |  |  | 18.6 |  |  | 17.3 |  |  | 93.4 |  |  | 1.3 |  |  | 0.31 |  |  |
| **Father education :** |  | 40.5 | <.001 |  | 49.1 | <.001 |  | 37.5 | <.001 |  | 2.3 | 0.072 |  | 61.0 | <.001 |  | 286.9 | <.001 |
| Low | 19.0 |  |  | 20.2 |  |  | 19.2 |  |  | 94.7 |  |  | 1.6 |  |  | -0.07 |  |  |
| Medium | 18.6 |  |  | 19.0 |  |  | 18.9 |  |  | 94.0 |  |  | 1.4 |  |  | 0.30 |  |  |
| High | 14.2 |  |  | 15.1 |  |  | 15.3 |  |  | 93.7 |  |  | 1.1 |  |  | 0.44 |  |  |
| Other-Uknw | 19.0 |  |  | 19.5 |  |  | 18.7 |  |  | 93.8 |  |  | 1.5 |  |  | 0.40 |  |  |
| **Mother education :** |  | 38.0 | <.001 |  | 45.9 | <.001 |  | 32.6 | <.001 |  | 2.1 | 0.100 |  | 69.5 | <.001 |  | 210.4 | <.001 |
| Low | 19.5 |  |  | 20.7 |  |  | 19.9 |  |  | 93.7 |  |  | 1.6 |  |  | -0.03 |  |  |
| Medium | 18.6 |  |  | 19.0 |  |  | 18.6 |  |  | 93.7 |  |  | 1.5 |  |  | 0.28 |  |  |
| High | 14.4 |  |  | 15.3 |  |  | 15.8 |  |  | 94.4 |  |  | 1.1 |  |  | 0.41 |  |  |
| Other-Uknw | 18.6 |  |  | 19.2 |  |  | 18.1 |  |  | 93.9 |  |  | 1.4 |  |  | 0.42 |  |  |
| **Subjective socio-economic ranking :** |  | 10.1 | <.001 |  | 8.9 | <.001 |  | 7.0 | <.001 |  | 3.5 | 0.008 |  | 7.6 | <.001 |  | 5.2 | <.001 |
| 5 or less | 18.2 |  |  | 18.8 |  |  | 18.2 |  |  | 94.5 |  |  | 1.4 |  |  | 0.12 |  |  |
| 6 | 17.8 |  |  | 18.5 |  |  | 18.4 |  |  | 94.4 |  |  | 1.3 |  |  | 0.05 |  |  |
| 7 | 16.7 |  |  | 17.9 |  |  | 17.6 |  |  | 93.7 |  |  | 1.4 |  |  | 0.06 |  |  |
| 8 | 16.4 |  |  | 17.5 |  |  | 17.3 |  |  | 93.3 |  |  | 1.4 |  |  | 0.09 |  |  |
| 9-10 | 18.9 |  |  | 17.9 |  |  | 17.4 |  |  | 94.4 |  |  | 1.2 |  |  | 0.13 |  |  |
| **Father working status :** |  | 11.6 | <.001 |  | 13.2 | <.001 |  | 17.5 | <.001 |  | 0.1 | 0.790 |  | 9.1 | 0.003 |  | 4359 | <.001 |
| Working | 17.2 |  |  | 17.9 |  |  | 17.6 |  |  | 94.0 |  |  | 1.4 |  |  | 0.66 |  |  |
| not working | 19.0 |  |  | 19.7 |  |  | 19.8 |  |  | 94.1 |  |  | 1.5 |  |  | -0.61 |  |  |
| **Mother working status :** |  | 0.1 | 0.813 |  | 0.0 | 0.862 |  | 0.5 | 0.496 |  | 0.0 | 0.904 |  | 9.3 | 0.002 |  | 2602 | <.001 |
| Working | 17.4 |  |  | 18.2 |  |  | 17.7 |  |  | 94.0 |  |  | 1.3 |  |  | 0.48 |  |  |
| not working | 17.3 |  |  | 17.9 |  |  | 18.0 |  |  | 93.9 |  |  | 1.4 |  |  | -0.33 |  |  |

Supplementary tables n°2. Comparison of socio-demographics distribution and smoking prevalences, SILNE 2013 survey and the HBSC 2009-010 survey.

| **Variables:** | SILNE 2013 survey** | HBSC 2009/2010 survey * |
| --- | --- | --- |
| Female (%) | 50.9 | 50.9 |
| Family affluence score (mean & std) | 5.6 (1.2) | 5.5 (1.3) |
| Age (y.) | 15.2 | 13.5 |
| Daily smoker (%) | 13.2 | 11.7 |
| Ever smoker (%) | 47.0 | 47.5 |

*computations made by MR ad IM, on the basis of HBSC 2009-2010 for Belgium, Finland, Germany, Italy , the Netherlands and Portugal, all age groups included.

**Weighted results.
